# Supplementary material for: Probabilistic dynamics of small groups in crowd flows
Source: PLoS One. 2026 Sep 10;21(9):e0356173. doi: 10.1371/journal.pone.0356173 (PMC13561383; doi:10.1371/journal.pone.0356173)
Supplement: Supplementary file 1 [file pone.0356173.s001.pdf]

S1 Appendix

Supplementary appendices for “Probabilistic dynamics of small groups in crowd flows” by Chiel van der Laan and Alessandro Corbetta.

DOI: 10.1371/journal.pone.0356173

A Definition of the standing threshold v_s

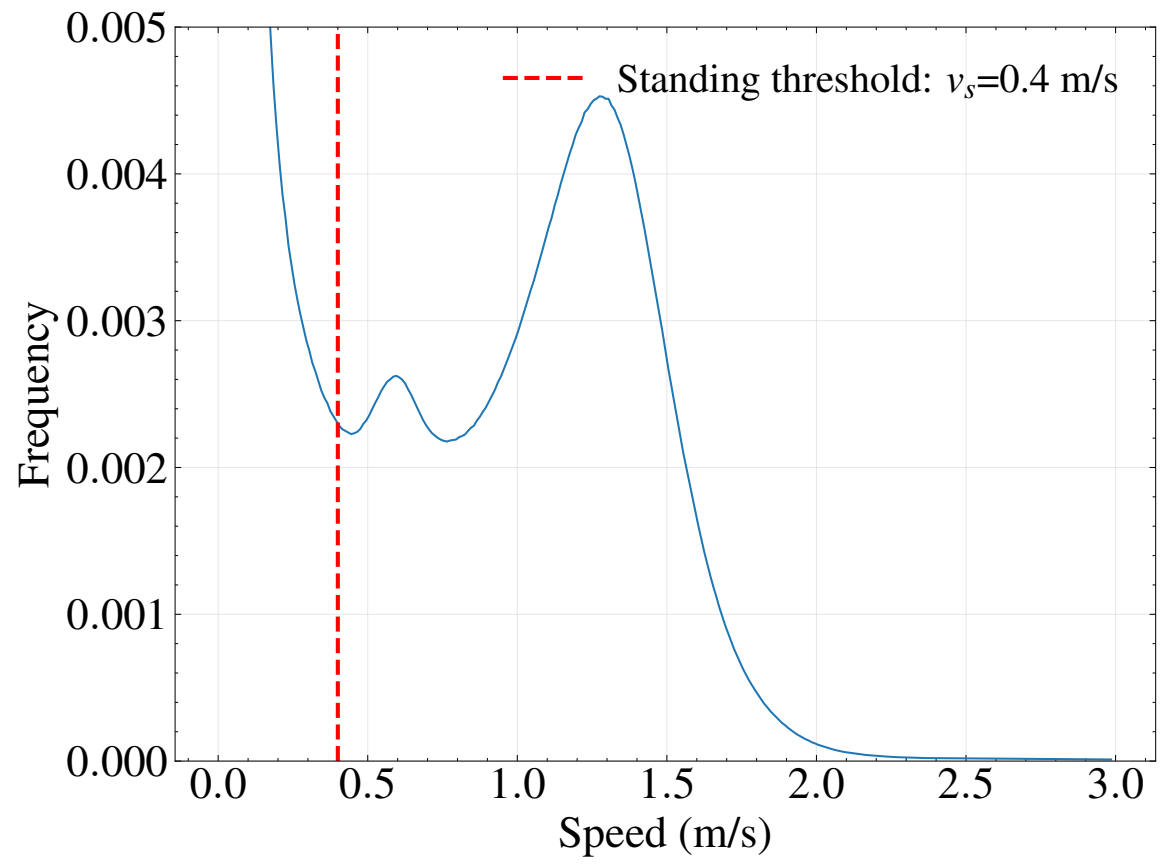

Fig A.1. Probability density function of pedestrian speeds from twenty randomly selected days in our dataset. The red dashed line marks the standing threshold $v_s = 0.4\,\mathrm{m/s}$, which is set between the stationary peak at $0.0\,\mathrm{m/s}$ and the slow-walking peak at $0.6\,\mathrm{m/s}$.

To distinguish between standing and moving pedestrians, we analyze the speed distribution of pedestrians in our dataset (Fig A.1). The distribution exhibits three distinct peaks: stationary pedestrians at $\approx 0.0\,\mathrm{m/s}$, slow walkers at $\approx 0.6\,\mathrm{m/s}$, and normal walkers at $\approx 1.3\,\mathrm{m/s}$. We set the standing threshold at $v_s = 0.4\,\mathrm{m/s}$, positioned between the stationary and slow-walking peaks.

B Dyad detection algorithm – technical details

The procedure described in Section 1.2 outlines the idealized detection scheme. In practice, to ensure only physically meaningful trajectory segments are analyzed, two imperfections in the raw dataset need to be addressed:

- **Detection failures.** A pedestrian may temporarily go undetected and reappear as a new trajectory, incorrectly causing connected components of size > 2 (see Fig B.1).
- **Boundary artifacts.** Velocities are unreliable near trajectory endpoints, requiring trimming of each co-observation interval.

These entail minor technical modifications of the dyad detection approach that we hereby describe.

Detection failures. Detection failures require special attention, because the procedure in Section 1.2 might unnecessarily reject valid dyads. For example, in the scenario of Fig B.1, pedestrian b is temporarily lost by the tracker and later re-identified as a new trajectory c. The procedure introduced in Section 1.2 would identify a, b, and c as part of a connected component of size 3, incorrectly discarding both valid pairs (a, b) and (a, c).

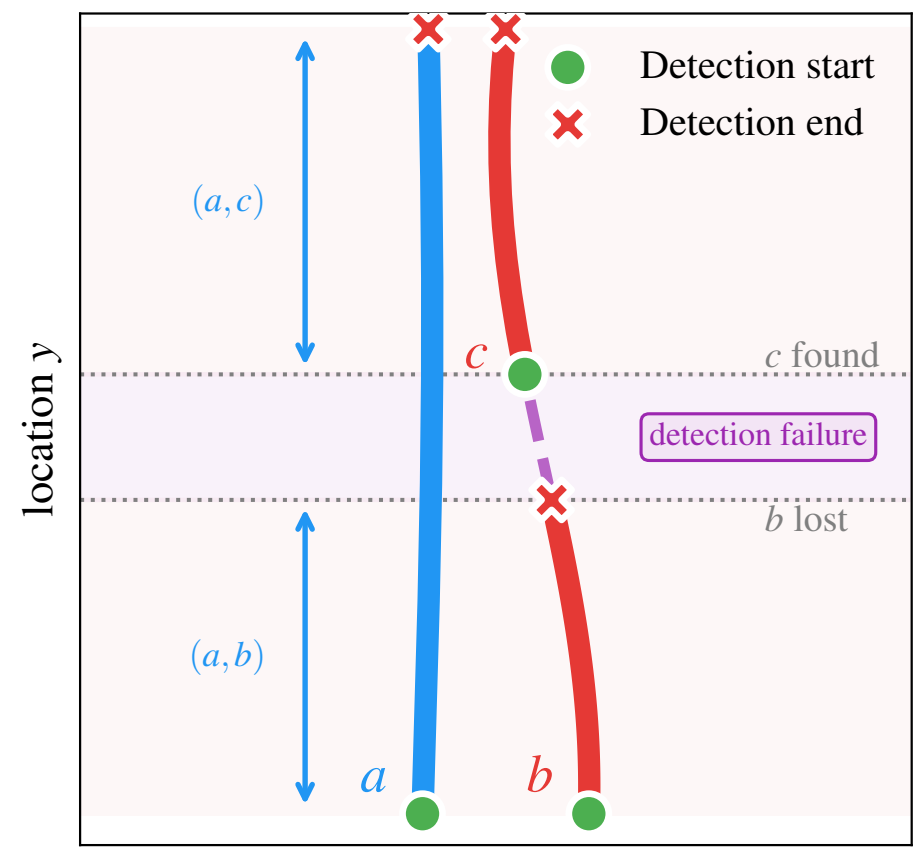

Fig B.1. Hardening the dyad detection algorithm in case of temporary tracking failures: example case. We sketch the trajectories, in blue and red, of a genuine dyad. The "blue" trajectory is correctly estimated and attributed identifier a. On the contrary, the tracker temporarily loses the "red" trajectory, resulting in distinct identifiers, b and c, appearing in disjoint time intervals. The idealized procedure in Section 1.2 would unnecessarily disregard similar cases. The technical workaround in Eq B.3 correctly retains these measurements.

To prevent such false rejections, we resolve dyad membership at each time step t rather than over the full trajectory. Concretely, we define the "active edge set" $\mathcal{E}_0(t)$ as

$$\mathcal{E}_0(t) = \{(\gamma_i(t), \gamma_j(t)) \mid (\gamma_i, \gamma_j) \in \mathcal{E}_0 \wedge t \in \mathcal{T}_i \cap \mathcal{T}_j\}\,. \tag{B.1}$$

This gives a snapshot of all co-present pairs at each time instant t. A genuine dyad member can be paired with at most one partner at any instant. We therefore mark a pedestrian as "ambiguous" at time t ($\mathcal{A}(t)$) if they are a member of more than one active candidate edge in $\mathcal{E}_0(t)$, i.e., their connected component is strictly larger than 2 or, in formulas,

$$\mathcal{A}(t) = \big\{k \;\big|\; \big|\{(\gamma_i(t), \gamma_j(t)) \in \mathcal{E}_0(t) : i = k \text{ or } j = k\}\big| > 1\big\}\,. \tag{B.2}$$

This allows us to replace the connected component pruning with a logic that retains only pairs from $\mathcal{C}$ in which neither partner is at any time "ambiguous". In equations, it holds:

$$\mathcal{C}_{\mathcal{A}} = \{(\gamma_i, \gamma_j) \in \mathcal{C} \mid \forall t :\; i \notin \mathcal{A}(t) \text{ and } j \notin \mathcal{A}(t)\}\,. \tag{B.3}$$

With this approach, both (a, b) and (a, c) from the case of Fig B.1 are retained as valid dyads.

Boundary artifacts. Velocity estimates derived exclusively from positional data become unreliable near the start and end of each trajectory. To exclude these unreliable segments, we trim the trajectory by the length of our Savitzky-Golay filter window Δt_{trim}. In equations, this trimmed co-observation interval $\mathcal{T}_{ij}^{\Delta}$ reads:

$$\mathcal{T}_{ij}^{\Delta} = \big[\min(\mathcal{T}_i \cap \mathcal{T}_j) + \Delta t_{\text{trim}},\; \max(\mathcal{T}_i \cap \mathcal{T}_j) - \Delta t_{\text{trim}}\big], \tag{B.4}$$

which, equivalently to Eq 17, results in the "trimmed walking time domain" $\tilde{\mathcal{T}}_{ij}^{\Delta}$:

$$\tilde{\mathcal{T}}_{ij}^{\Delta} = \big\{\, t \in \mathcal{T}_{ij}^{\Delta} \;\big|\; \|\mathbf{v}_i(t)\| > v_s,\; \|\mathbf{v}_j(t)\| > v_s \,\big\}. \tag{B.5}$$

The trimming necessitates a modification to $\mathcal{D}$ (cf. Eq 20) that now has to satisfy three criteria: (i) to discard excessively short trajectories, as in Eq 20, the co-observation interval must be longer

than $t_{\min}$; (ii) to ensure we did not trim away only parts where the dyad was walking, the trimmed trajectories must at least be walking for $\tilde{t}^{\Delta}_{\min}$; and (iii) to check for proximity in this new domain, the mean inter-pedestrian distance during $\tilde{\mathcal{T}}^{\Delta}_{ij}$ must be smaller than d_{max}. The modified equation thus reads

$$\mathcal{D} = \left\{ (\gamma_i, \gamma_j) \in \mathcal{C}_{\mathcal{A}} \;\middle|\; \begin{array}{l} |\mathcal{T}_{ij}| > t_{\min} \\ |\tilde{\mathcal{T}}^{\Delta}_{ij}| > t^{\Delta}_{\min} \\ \langle d_{ij} \rangle_{\tilde{\mathcal{T}}^{\Delta}_{ij}} < d_{max} \end{array} \right\}, \qquad \begin{array}{l} t_{\min} = 8.0 \text{ s} \\ t^{\Delta}_{\min} = 4.0 \text{ s} \\ d_{max} = 1.5 \text{ m} \\ \Delta t_{\text{trim}} = 2.2 \text{ s.} \end{array} \tag{B.6}$$

C Sensitivity analyses

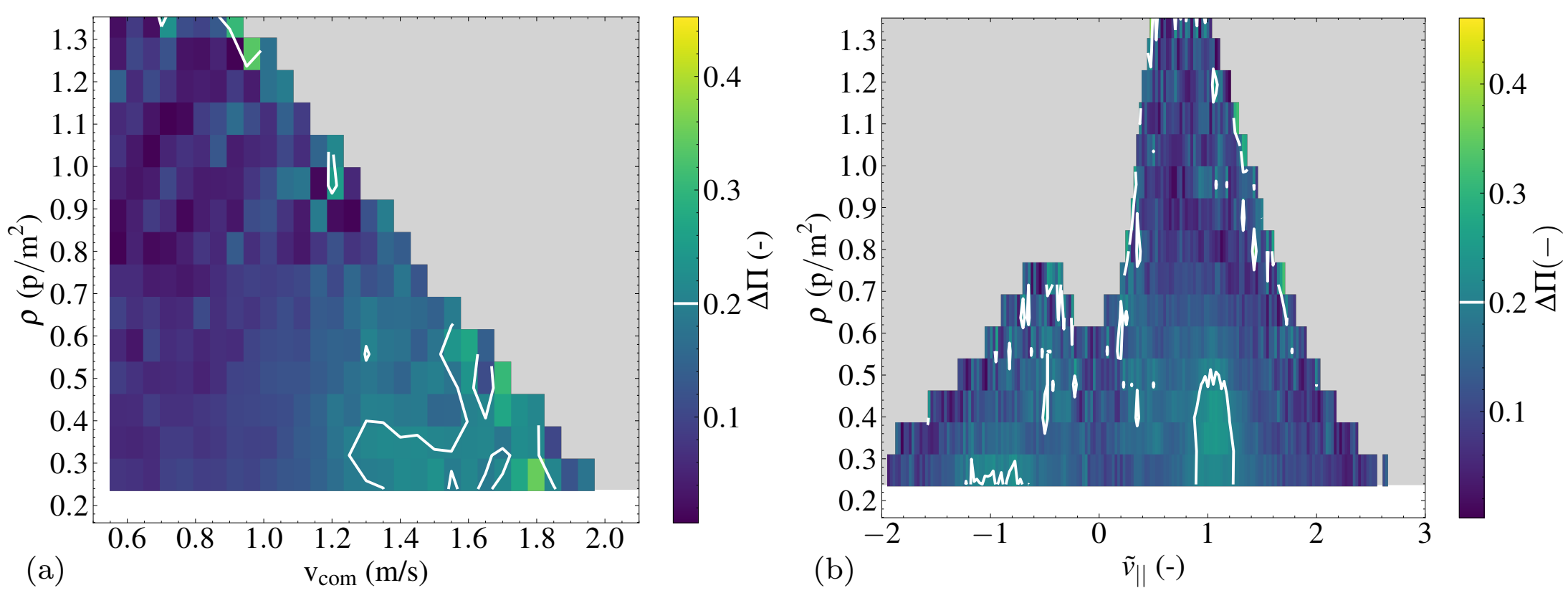

Fig C.1. Sensitivity of the empirical formation indicator Π_{OLO} to a joint $\pm 10\%$ perturbation of the dyad detection parameters in Eq B.6, relative to the original parameter set though $\Delta\Pi$ (cf. Eq C.1). (a) $\Delta\Pi$ for $\Pi^{\text{standing}}_{\text{OLO}}(\text{v}_{\text{com}}, \rho)$ (cf. Fig 8c), which remains consistently lower than 0.2 (with Π_{OLO} typically having order 1). (b) $\Delta\Pi$ for $\hat{\Pi}_{\text{OLO}}(\tilde{v}_{||}, \rho)$ (cf. Fig 10) which also remains typically well below 0.2.

We assess the robustness of our dyad detection algorithm and our overall findings with respect to $\pm 10\%$ perturbations of key parameters:

- the detection thresholds $t_{\min}$, $t^{\Delta}_{\min}, d_{max}$ that determine which pairs are retained as dyads.
- the radius R that governs local density estimation.

We show that our results are practically insensitive to these perturbations.

Robustness of the detected dyad set. We verify that the detected dyad set is insensitive to the precise choice of the three detection thresholds $t_{\min}$, $t^{\Delta}_{\min}$, and d_{max} (cf. Eq 19, Eq B.6) by applying a joint $\pm 10\%$ perturbation, yielding the parameter and related dyad sets:

- -10% perturbation (dyad set $\mathcal{D}_-$): $t_{\max} = 7.0\,\text{s}$, $\tilde{t}_{\max} = 3.3\,\text{s}$, and $d_{\max} = 1.4\,\text{m}$;
- original parameters (dyad set $\mathcal{D}$): $t_{\max} = 8.0\,\text{s}$, $\tilde{t}_{\max} = 4.0\,\text{s}$, and $d_{\max} = 1.5\,\text{m}$;
- $+10\%$ perturbation (dyad set $\mathcal{D}_+$): $t_{\max} = 8.8\,\text{s}$, $\tilde{t}_{\max} = 4.4\,\text{s}$, and $d_{\max} = 1.6\,\text{m}$.

As reported in Table C.1, the dyad set $\mathcal{D}$ has more than 83% overlap (in terms of data samples, indicated by the operator $|\cdot|$) with the perturbed sets $\mathcal{D}_+$ and $\mathcal{D}_-$. Most importantly, these differences in dyad selection account to no practical distinction when it comes to quantify the statistical properties of the dyads and our models thereof.

Table C.1. Pairwise relative overlap between dyad sets detected under the original parameter set ($\mathcal{D}$) and under the ±10% perturbed parameter sets ($\mathcal{D}_-$ and $\mathcal{D}_+$).

Relative overlap (%)		Jaccard (%)
$\lvert\mathcal{D}\cap\mathcal{D}_-\rvert/\lvert\mathcal{D}\rvert = 86\%$	$\lvert\mathcal{D}\cap\mathcal{D}_-\rvert/\lvert\mathcal{D}_-\rvert = 87\%$	$\lvert\mathcal{D}\cap\mathcal{D}_-\rvert/\lvert\mathcal{D}\cup\mathcal{D}_-\rvert = 80\%$
$\lvert\mathcal{D}\cap\mathcal{D}_+\rvert/\lvert\mathcal{D}\rvert = 89\%$	$\lvert\mathcal{D}\cap\mathcal{D}_+\rvert/\lvert\mathcal{D}_+\rvert = 83\%$	$\lvert\mathcal{D}\cap\mathcal{D}_+\rvert/\lvert\mathcal{D}\cup\mathcal{D}_+\rvert = 80\%$
$\lvert\mathcal{D}_-\cap\mathcal{D}_+\rvert/\lvert\mathcal{D}_-\rvert = 87\%$	$\lvert\mathcal{D}_-\cap\mathcal{D}_+\rvert/\lvert\mathcal{D}_+\rvert = 79\%$	$\lvert\mathcal{D}_-\cap\mathcal{D}_+\rvert/\lvert\mathcal{D}_-\cup\mathcal{D}_+\rvert = 73\%$

To confirm that this robustness translates to the main results, Fig C.1 reports the pointwise absolute differences with respect to the original values of $\Pi^{\text{standing}}_{\text{OLO}}(\text{v}_{\text{com}}, \rho)$ and $\hat{\Pi}_{\text{OLO}}(\tilde{v}_{||}, \rho)$ under all three parameter sets. Specifically, we consider the indicator

$$\Delta\Pi(\mathbf{v}_{com}, \rho) = \max\left\{\lvert\Pi_{\mathcal{D}} - \Pi_{\mathcal{D}_-}\rvert, \lvert\Pi_{\mathcal{D}} - \Pi_{\mathcal{D}_+}\rvert\right\}. \tag{C.1}$$

The empirical values have with maximum pointwise deviation from the original below 0.2 across large parts of the domain.

Sensitivity of density-dependent analyses to R. The radius R (cf. Eq 11) determines the local density ρ used in all analyses. By construction, R does not affect the dyad identification procedure. We demonstrate that ±10% perturbations in R reflect into predictable shifts in ρ. Our density definition (cf. Eq 11) can be decomposed into a crowd and a dyad contribution:

$$\rho = \frac{N_R}{\pi R^2} = \frac{N_R - 2}{\pi R^2} + \frac{2}{\pi R^2} = \rho_{\text{crowd}}(R) + \rho_{\text{free}}(R), \tag{C.2}$$

where, under the ±10% variations, $\rho_{\text{crowd}} = \rho - \rho_{\text{free}}$ is effectively insensitive to changes in R. In equations, for any perturbed radius R' and original radius R, it holds:

$$\rho(R') - \rho_{\text{free}}(R') \approx \rho(R) - \rho_{\text{free}}(R). \tag{C.3}$$

Therefore, the aforementioned predictable shift for a perturbed radius R' with respect to our original choice R is:

$$\rho(R') \approx \rho(R) - \rho_{\text{free}}(R) + \rho_{\text{free}}(R') = \frac{N_R}{\pi R^2} - \frac{2}{\pi R^2} + \frac{2}{\pi R'^2}. \tag{C.4}$$

Figure C.2 confirms the validity of this shift for both $\Pi^{\text{standing}}_{\text{OLO}}(\text{v}_{\text{com}}, \rho)$ and $\hat{\Pi}_{\text{OLO}}(\tilde{v}_{||}, \rho)$ by plotting three contours as a function of ρ_{crowd} instead of ρ. As one can observe, there is near perfect overlap, reinforcing the weak sensitivity on R.

D Modal distance definition

To extract characteristic interpersonal distances for each configuration type (abreast and in-file), we integrate the probability distributions $\mathbb{P}(x_r, y_r)$ over narrow bands around each principal axis (Fig D.1a depicts the integration domains). In formulas, let

$$g^{\leftrightarrow}(x_r) = \int_{-\Delta b}^{\Delta b} \mathbb{P}(x_r, y_r)\, dy_r, \quad g^{\updownarrow}(y_r) = \int_{-\Delta b}^{\Delta b} \mathbb{P}(x_r, y_r)\, dx_r \tag{D.1}$$

where $\Delta b = 0.15\,\text{m}$ defines the bandwidth. As illustrated in Fig D.1b, we fit each marginal distribution with a Gaussian:

$$G(\ell) \propto \exp\left(-\frac{(\ell - \mu)^2}{2\sigma^2}\right), \tag{D.2}$$

where μ is the modal position and σ is the standard deviation. Finally, we define the interpersonal distance as twice the value of μ, as in Eq 23 (cf. Fig D.1b).

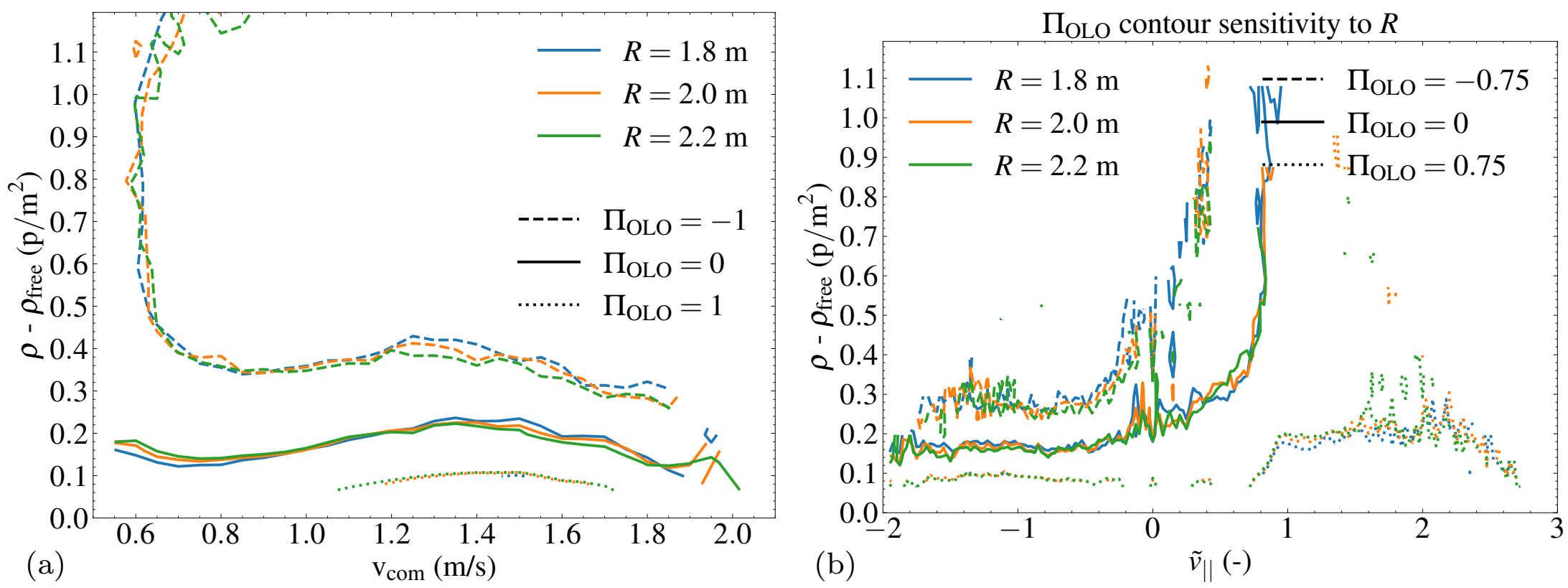

Fig C.2. Sensitivity of empirical Π_{OLO} values to a $\pm 10\%$ variation of the density radius R in Eq 11. Contours computed with $R = 1.8\,\mathrm{m}$, $R = 2.0\,\mathrm{m}$ (as in Section 1.1), and $R = 2.2\,\mathrm{m}$ are shown in each figure as blue lines, orange lines, and green lines, respectively. For each radius, three contour levels are displayed using solid, dashed, and dotted line styles. (a) Three $\Pi_{\mathrm{OLO}}^{\mathrm{standing}}(\mathrm{v}_{\mathrm{com}}, \rho_{\mathrm{crowd}})$ contours for the three radii, showing substantial overlap. (b) Three $\hat{\Pi}_{\mathrm{OLO}}(\tilde{v}_{||}, \rho_{\mathrm{crowd}})$ contours, also exhibiting strong overlap. These figures indicate that the observed differences are primarily due to a predictable shift along the density axis, as in Eq C.4.

E Region of validity for the Π_{OLO} model

In Section 3.2 and Section 3.3.1, we provided phenomenological models for Π_{OLO} in standing crowd and counter-flow conditions, respectively. These models are defined only in regions where sufficient data is available. Specifically, the model for $\Pi_{\mathrm{OLO}}^{\mathrm{standing}}(\mathrm{v}_{\mathrm{com}}, \rho)$ (Eq 44) has validity in the region

$$\{(\mathrm{v}_{\mathrm{com}}, \rho) \colon \rho_{\mathrm{free}} = 0.16\,\mathrm{p/m}^2 < \rho < A\,\mathrm{v}_{\mathrm{com}} + B, \quad \mathrm{v}_{\mathrm{com}} > v_s\}, \tag{E.1}$$

where $A = -1.19\,\mathrm{ps/m}^3$, $B = 2.49\,\mathrm{p/m}^2$. Whereas the model for $\Pi_{\mathrm{OLO}}^{\mathrm{counterflow}}(\mathrm{v}_{\mathrm{com}}, \rho)$ (Eq 49) has validity in

$$\{(\mathrm{v}_{\mathrm{com}}, \rho) \colon \rho_{\mathrm{free}} < \rho < C\,\mathrm{v}_{\mathrm{com}}^2 + D\,\mathrm{v}_{\mathrm{com}} + E, \quad \mathrm{v}_{\mathrm{com}} > v_s\}, \tag{E.2}$$

where $C = -0.35\,\mathrm{ps}^2/\mathrm{m}^4$, $D = 0.51\,\mathrm{ps/m}^3$, and $E = 0.53\,\mathrm{p/m}^2$. In Fig E.1, we illustrate these valid regions.

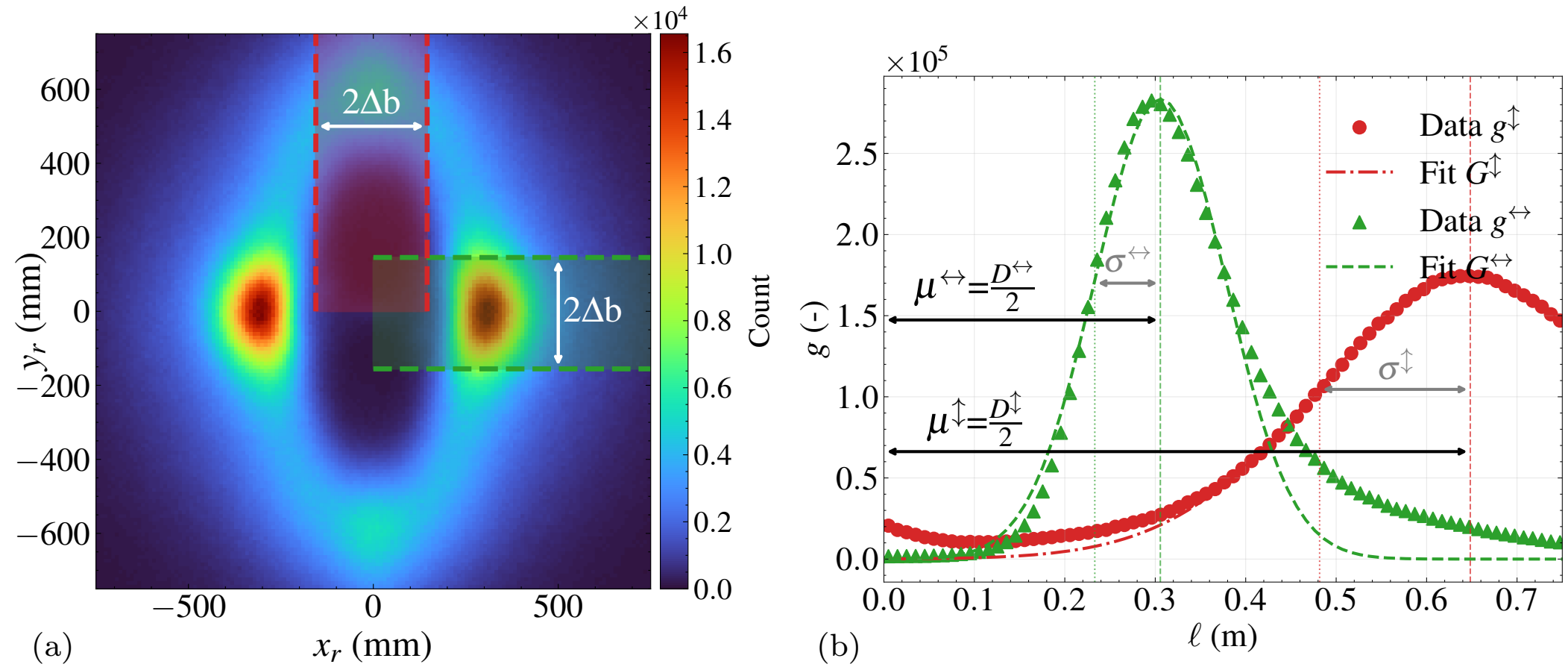

Fig D.1. (a) Probability distribution $\mathbb{P}(x_r, y_r)$, the bands in green and red indicate the integration domains for $g^{\leftrightarrow}(x_r)$ and $g^{\updownarrow}(y_r)$ respectively (Eq D.1). (b) The data points retrieved from the integrations for $g^{\leftrightarrow}$ in green triangles and $g^{\updownarrow}$ in red circles with their corresponding G fits.

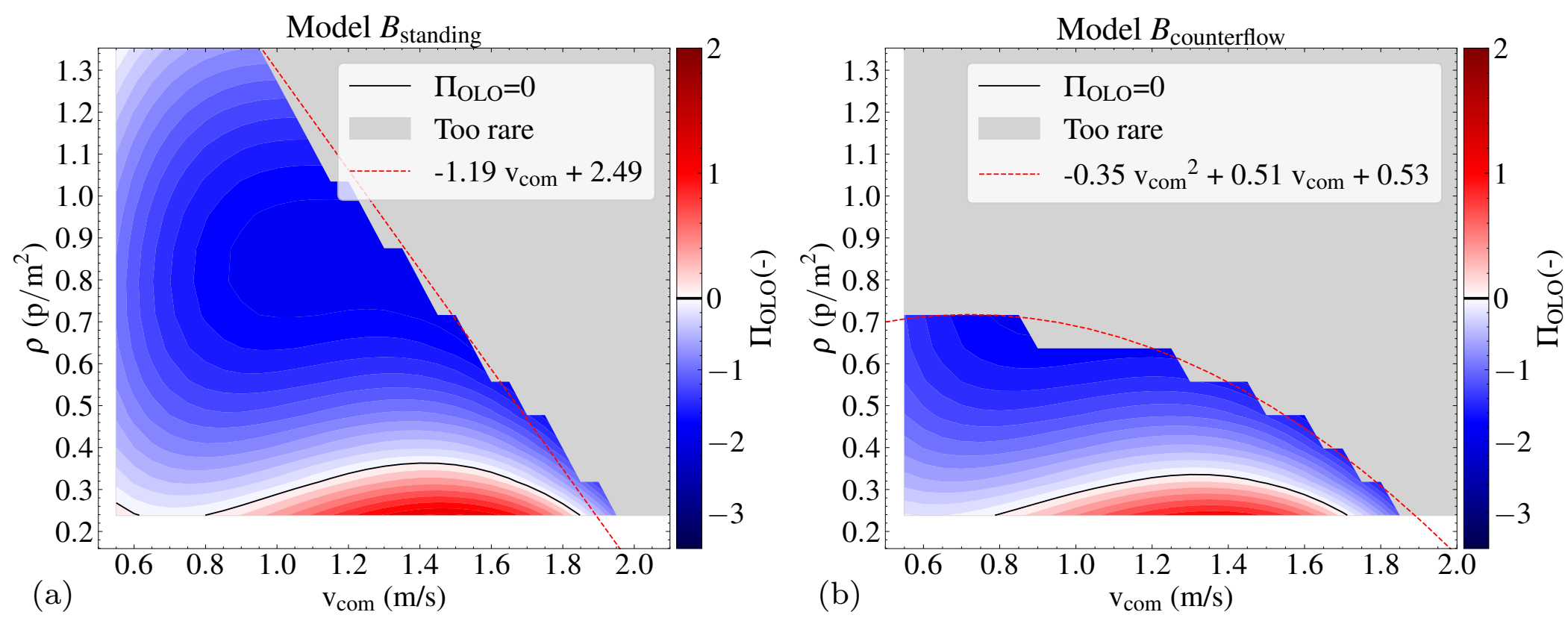

Fig E.1. Models for $\Pi_{\text{OLO}}^{\text{standing}}(\text{v}_{\text{com}}, \rho)$ (Eq 44) and $\Pi_{\text{OLO}}^{\text{counterflow}}(\text{v}_{\text{com}}, \rho)$ (Eq 49), with their respective validity regions. (a) Standing crowd model (Eq 44) with validity boundary (dashed red line, Eq E.1). (b) Counter-flow model (Eq 49) with validity boundary (dashed red line, Eq E.2). Gray regions indicate insufficient data for reliable model predictions.

F Robustness of the Π_{OLO} model parameters

To assess the robustness of the fitted parameters $\text{v}_L(\rho)$ and $\text{v}_H(\rho)$ (cf. Section 3.2 and Table 1), we perform a 5-fold split-sample validation at the trajectory level. The 900-day dataset is partitioned randomly into five non-overlapping subsets of 180 days each. For each of these folds, $s = 1, \ldots, 5$, the complete pipeline (dyad detection, binning, and model fitting) is executed independently. The spread across folds is shown as asymmetric error bars, with the lower and upper extents given by

$$\sigma^-_{\text{v}_L}(\rho_k) = \bar{v}_L(\rho_k) - \min_s \text{v}_L^{(s)}(\rho_k), \qquad \sigma^+_{\text{v}_L}(\rho_k) = \max_s \text{v}_L^{(s)}(\rho_k) - \bar{v}_L(\rho_k), \tag{F.1}$$

where $\bar{v}_L(\rho_k) = \frac{1}{5}\sum_s \text{v}_L^{(s)}(\rho_k)$ is the mean across folds, and analogously for v_H. Fig F.1 shows the mean and error bars for the empirical v_L and v_H values across folds, together with the fitted curves from the full dataset. The narrow error bars for v_L indicate that these parameters are robustly identified across folds. For v_H, the error bars are wider, likely reflecting greater data variability in these regimes.

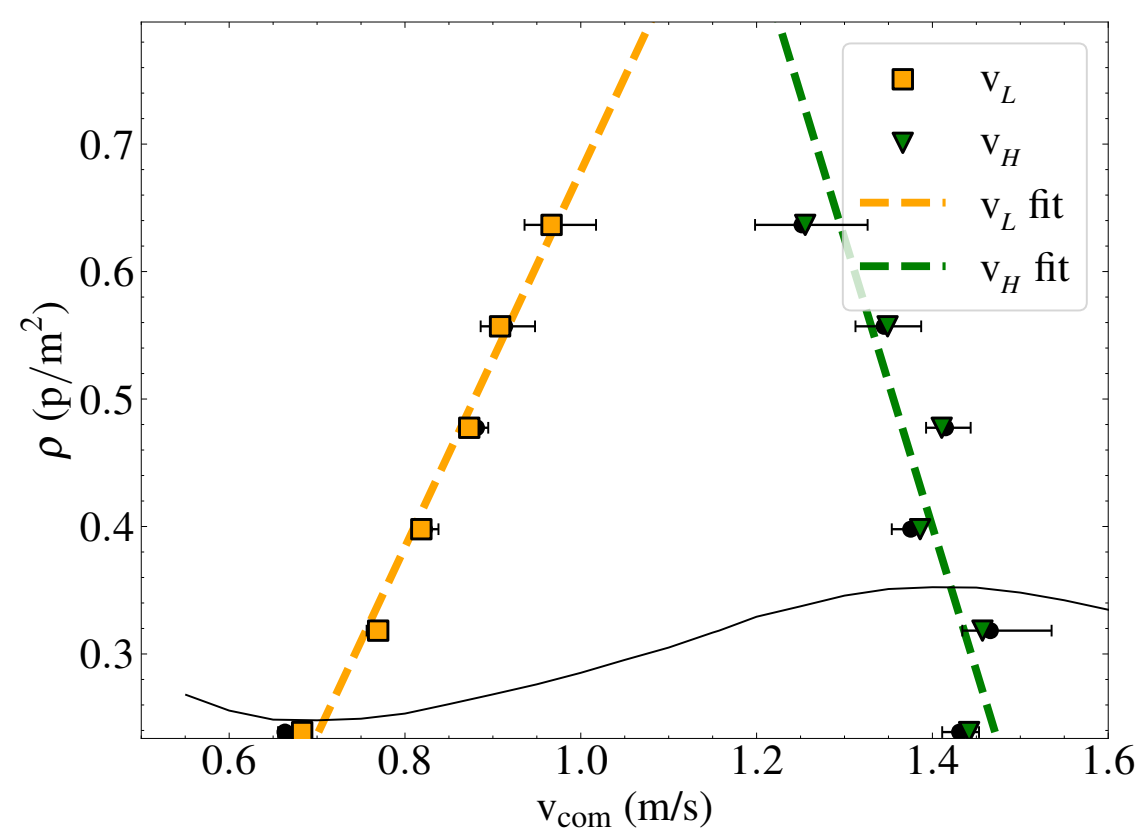

Fig F.1. Split-sample validation of the parameters $v_L(\rho)$ and $v_H(\rho)$ for the standing crowd model (cf. Eq 44). The empirical v_L and v_H (respectively, yellow squares and green triangles) are shown along with their corresponding fits (dashed lines). In black the empirical split-sample mean (circles) and error bars (cf. Eq F.1) are overlaid, with a black contour line that marks $\Pi_{\text{OLO}} = 0$ from Fig 8c as a reference.
